# Simultaneous Electrochemical Deposition of Cobalt Complex and Poly(pyrrole) Thin Films for Supercapacitor Electrodes

**DOI:** 10.1038/s41598-019-41969-6

**Published:** 2019-04-04

**Authors:** Charlette M. Parnell, Bijay P. Chhetri, Travis B. Mitchell, Fumiya Watanabe, Ganesh Kannarpady, Ambar B. RanguMagar, Huajun Zhou, Karrer M. Alghazali, Alexandru S. Biris, Anindya Ghosh

**Affiliations:** 1 Department of Chemistry, University of Arkansas at Little Rock, 2801 South University Avenue, Little Rock, AR 72204 USA; 2 Center for Integrative Nanotechnology Sciences, University of Arkansas at Little Rock, 2801 South University Avenue, Little Rock, AR 72204 USA; 3 High-Density Electronics Center, University of Arkansas, Fayetteville, AR 72701 USA

## Abstract

Supercapacitors are beneficial as energy storage devices and can obtain high capacitance values greater than conventional capacitors and high power densities compared to batteries. However, in order to improve upon the overall cost, energy density, and charge-discharge rates, the electrode material of supercapacitors needs to be fine-tuned with an inexpensive, high conducting source. We prepared a Co(III) complex and polypyrrole (PPy) composite thin films (CoN_4_**-**PPy) that was electrochemically deposited on the surface of a glassy carbon working electrode. Cyclic voltammetry studies indicate the superior performance of CoN_4_-PPy in charge storage in acidic electrolyte compared to alkaline and organic solutions. The CoN_4_-PPy material generated the highest amount of specific capacitance (up to 721.9 F/g) followed by Co salt and PPy (Co-PPy) material and PPy alone. Cyclic performance studies showed the excellent electrochemical stability of the CoN_4_-PPy film in the acidic medium. Simply electrochemically depositing an inexpensive Co(III) complex with a high electrically conducting polymer of PPy delivered a superior electrode material for supercapacitor applications. Therefore, the results indicate that novel thin films derived from Co(III) metal complex and PPy can store a large amount of energy and maintain high stability over many cycles, revealing its excellent potential in supercapacitor devices.

## Introduction

The widespread global energy consumption is slowly depleting our limited resources. With most of the energy production manufactured from non-renewable resources such as fossil fuels, it is essential to investigate alternative energy sources that are clean, efficient and renewable^1^. There are several types of renewable energy sources such as solar and wind energy that have been beneficial in providing energy to meet our needs^2,3^. However, to sufficiently provide energy during times of no sunlight or wind, a device that harvests and stores the energy needs to be implemented. Some examples of such devices that have been employed in this regard include batteries, capacitors and supercapacitors^4–6^. Compared to batteries, supercapacitors can deliver charge quickly for higher power density^7^. There are two types of supercapacitors: electrochemical double layer capacitors (EDLCs) and pseudocapacitors. The former non-Faradaically stores charge with an electrochemical double-layer while the latter uses Faradaic redox processes, electrosorption and/or intercalation processes^8^. The efficiency of supercapacitors lies within the electrode material as this can dramatically influence the surface area, conductivity and overall resistance of the device. Moreover, the cost of electrode materials hinders the widespread use of these devices^9^. Therefore, it is important to develop electrode materials that couple high capacitance (to improve charge-discharge cycling) with reduced material cost^10^. Liu *et al*. developed hollow, spherical nitrogen-rich porous carbon shells *via* carbonization of porous organic frameworks (POFs) to be used as an electrode material in a supercapacitor^11^. High capacitance up to 230 F/g was achieved at current density of 0.5 A/g with excellent cycling performance up to 1500 cycles. While designing POF electrodes in supercapacitor devices can give better electrochemical stability in these materials, the capacitance still needs to be improved to meet the standards of commercialization of these devices.

Deviating from porous controllability, use of transition metal oxides such as NiCo_2_O_4_^12^, MoO_3_^13^, and MnO_2_^14^ have been investigated as pseudocapacitive materials for supercapacitors owing to their low cost, different oxidation states, low toxicity, high power output, and high capacitance^15,16^. As for example, Lei *et al*. synthesized NiCo_2_O_4_ by rapid and template-free microwave-assisted heating reflux method to fabricate electrode materials for supercapacitors. These flower-shaped microspheres possess large specific surface area, which assisted in its high specific capacitance of 1006 F/g. Even after 1000 electrochemical cycles, the material retained 93.2% of its activity at current density of 8 A/g^12^. In the search of new class of electrode materials for supercapacitors, transition metal sulfides have also been investigated^16,17^. Shen *et al*. reported synthesis of NiCo_2_S_4_ nanosheets grown on nitrogen-doped carbon foams (NiCo_2_S_4_/NCF), which exhibited a ultrahigh capacitance of 1025 F/g at 10 A/g with excellent electrochemical cycling stability with only 9.6% loss of performance after 2000 cycles at 10 A/g^17^. Thus, a new approach incorporating inexpensive metals has shown to possess promising potential for electrochemical supercapacitors. Incorporating this idea and renewable materials, the performance and cost of supercapacitors and their electrode materials can be further improved.

Aside from investigating inexpensive metals, conducting polymers such as polyaniline (PANI)^18^, polythiophene (PTh)^19^, and polypyrrole (PPy)^20^ have gained much attention throughout the scientific community as materials that can enhance capacitance efficiency for charge storage^21,22^. Among different conducting polymers, PPy has shown to be the most promising due to its high conductivity, low cost, and ease of synthesis. Moreover, PPy exhibits excellent redox reversibility and environmental stability^23^. There are two methods of PPy synthesis: chemical and electrochemical. Of the two synthetic routes, electrochemical deposition provides samples that are more conductive and exhibit a higher capacitance than those developed from chemical methods^24^. While several metal oxides and metal sulfides have been used to design electrode materials for fuel cells and supercapacitors, the use of metal complexes has been relatively low. Over the past few decades, extensive efforts have been made to design catalytic materials bearing macrocyclic metal N_4_ complexes such as porphyrin and phthalocyanines^25,26^. The macrocyclic metal N_4_ complexes with different transition metals have been identified as a promising electrode material for fuel cells^27–29^ and lithium-air (Li_2_O_2_) battery^30^. Recently, we demonstrated the use of macrocyclic Co(III)^31^ and Mn(III) N_4_ complexes coated with polydopamine^32^ and supported on graphene as a promising electrocatalyst for oxygen reduction reaction (ORR). In the past, research on metal N_4_ complexes has mainly focused on the electrochemical ORR and covered less on supercapacitors. Thus, we extended our work on Co(III) N_4_ macrocyclic complex for supercapacitors.

**Figure 1:**
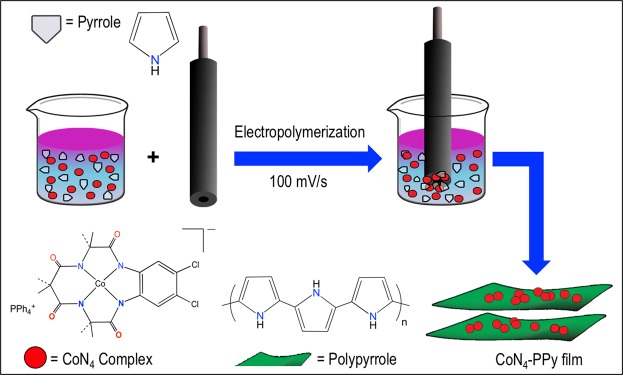
Schematic representation of electrochemical deposition of PPy films using pyrrole and CoN_4_ complex. The films were deposited on GCE.

In this manuscript, we report the electrochemical deposition of PPy films onto a glassy carbon electrode (GCE). To increase the capacitance, Co metal impurities in the forms of either Co-amidomacrocyclic metal catalyst (CoN_4_) or Co acetate salt, were integrated into the film network (Fig. 1). Of these impurities used, CoN_4_ facilitated greater supercapacitive activity with a high specific capacitance up to 721.9 F/g. Galvanostatic charge-discharge (GCD) cycles were performed and revealed high rate of charge-discharge for CoN_4_-PPy films. Furthermore, CoN_4_-PPy films provided excellent electrochemical stability in acidic media as revealed by cyclic performance studies. Therefore, the use of Co(III) complex greatly enhanced the specific capacitance of PPy films. To the best of our knowledge, we are the first to develop a conductive electrode film using macrocyclic Co(III)N_4_ complexes and conductive polymer (PPy) by electrodeposition which demonstrates superior supercapacitor performance. Our CoN_4_-PPy film offers an exceptional platform for generating novel and inexpensive electrode materials and could be a significant improvement in the field of supercapacitors.

## Results and Discussion

### Synthesis and Characterization of CoN_4_-PPy film

**Figure 2:**
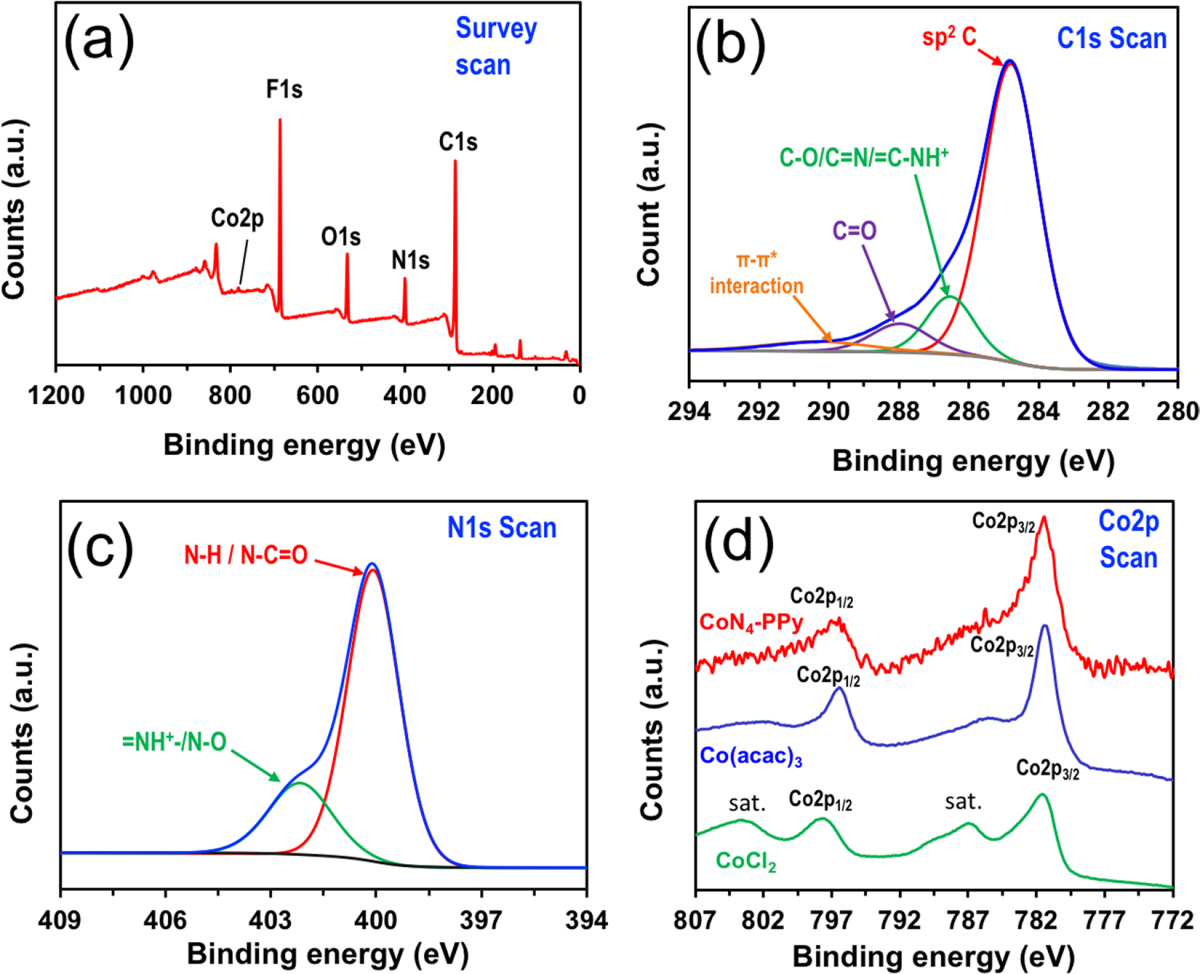
(**a**) XPS survey scan of CoN_4_-PPy film, and a representative XPS narrow scans for (**b**) Carbon (C1s), (**c**) Nitrogen (N1s), and (**d**) Cobalt (Co2p) present in CoN_4_-PPy film. Figure (**d**) also shows Co2p spectra of CoCl_2_ and (Co(acac)_3_) as reference samples.

A scheme for the synthesis of CoN_4_**-**PPy film is shown in Fig. 1. Once synthesized, X-ray photoelectron spectroscopy (XPS) was performed to identify the elemental composition, bond formation, and the oxidation state of Co metal in the thin film as shown in Fig. 2.

The survey scan (Fig. 2a) shows the co-existence of C, N, O, and Co, the content of which are provided in Supplementary Table S1. F signal comes from the residual anion of PF_6_^−^ (hexafluorophosphate anion). The narrow scan of C1s spectrum (Fig. 2b) shows several peaks over the range of 284.8 to 289.1 eV. A strong dominant peak at 284.8 eV is assigned to sp^2^ hybridized carbon present in aromatic ring of PPy^33^. This peak also belongs to carbon atoms present in the aromatic ring and other positions of CoN_4_ complex. Other carbon peaks at binding energy of 286.5 eV and 287.9 eV indicate the possible formation of C-OH/C=N/=C-NH^+^ and C=O in the CoN_4_**-**PPy film, respectively^33^. The peak positioned at highest binding energy of 290.6 eV can be assigned to π-π* interaction arising from the aromatic rings of PPy or CoN_4_ complex^33,34^. Nitrogen peak (Fig. 2c) located at 400.1 eV is related to –NH- bond and amide linkage (N-C=O) present in PPy and CoN_4_ complex, respectively^33,35^. This peak also indicates a contribution from four chemically equivalent nitrogen atoms bound to the metal center (N-Co) in CoN_4_ complex^36,37^. Further, a peak at higher binding energy value of 402.2 eV is indicative of =NH^+^-/N-O bonds^33,37^. Similar peaks were seen in the XPS narrow scans of C1s and N1s of PPy film (Supplementary Fig. S1a,b). Further, Co2p narrow scan of CoN_4_-PPy film revealed two major peaks. The first peak that appeared at 781.8 eV is assigned to Co2p_3/2_ and is most likely Co-N_x_ bond in CoN_4_ complex^38^. The second peak at 796.9 eV is ascribed to Co2p_1/2_. The peak separation between these two peaks was approximately 15.1 eV, which is indicative of Co species in +3 oxidation state^39^. To confirm the existence of +3 oxidation state in CoN_4_-PPy thin film, we performed the XPS of Cobalt(II) chloride (CoCl_2_) and Cobalt(III)acetylacetonate (Co(acac)_3_). The shape of Co 2p spectra of these two reference samples were compared with the shape of the Co 2p spectra in CoN_4_-PPy thin film (Fig. 2d). Similar peak shapes observed in Co2p spectra both for CoN_4_-PPy and Co(acac)_3_ indicate that the Co in CoN_4_-PPy thin film is indeed in +3 oxidation state. Furthermore, two distinct satellite peaks, characteristic for Co in the +2 oxidation state, were displayed in the Co 2p spectra of CoCl_2_. These satellite peaks were not observed in the CoN_4_-PPy thin film, which is indicative of Co in the +3 oxidation state in CoN_4_-PPy thin film.

Further, to monitor any changes in the chemical nature of C, N, and Co after exposure to the highly acidic environment (HClO_4_) that was used during the supercapacitor performance, XPS of CoN_4_**-**PPy film was also conducted. For an acid exposed CoN_4_**-**PPy film, the C1s narrow scan indicated slight shifting of the peaks at 286.5, 287.9, and 290.6 eV to 286.4, 288.6, and 290.3 eV, respectively (Supplementary Fig. S2a). Likewise, the N1s narrow scan show slight shifting of the peaks (Supplementary Fig. S2b). The shifting of these peaks may be due to some mathematical random fluctuation during XPS fitting. Any changes with the Co2p narrow scan would show possible demetallation of Co from the metal complex. Also, shifting in the peak positions, peak separation, and appearance of satellite peaks would indicate any potential changes of the Co metal oxidation in the complex. However, none of these phenomena were observed in the XPS of Co2p narrow scan for the acid exposed CoN_4_-PPy film. The peak separation between Co2p_1/2_ and Co2p_3/2_ remained to be 15.0 eV indicating Co(III) oxidation state (Supplementary Fig. S2c)^39^. Therefore, it can be inferred from the XPS analysis that the Co metal in CoN_4_ is stable within the acidic electrolyte which may be attributed to its strong attachment with PPy. This attachment could facilitate the reaction between Co and electrolyte, and thus results in a higher capacitance.

Raman spectroscopy was also used to evaluate the stability of CoN_4_-PPy films after exposure in acidic solution (Supplementary Fig. S3). The two intense peaks at 1340 and 1570 cm^−1^ correspond with the C-N and C=C stretching frequencies present in PPy, respectively^40^. An additional peak at 1050 cm^−1^ is characteristic of C-H in-plane deformation of PPy^41^. A small range Raman shift was performed to observe the presence of the Co catalyst in the film. A distinct broader peak at 405 cm^−1^ could be attributed to the Co-N stretch^42^. This is slight shift from the 420 cm^−1^ peak observed in a previously synthesized Co(III)-graphene nanocomposite, which could be from the interaction of the Co catalyst with PPy^40^. After exposure to acidic electrolyte, Raman spectroscopy was conducted and revealed no appearance of a band between 3200–3300 cm^−1^, which would indicate N-H stretching frequency present in ligand (N_4_). Therefore, it can be inferred that the Co(III) complex in CoN_4_-PPy films is relatively stable in acidic environment even though Co(III) salt is susceptible to dissolve in acidic medium, which may undergo reduction to lower oxidation state such as Co(II). This result was illustrated by our findings with XPS. The perfect morphology of as-synthesized CoN_4_-PPy thin film, high stability of metal N4 complexes, and greater attachment of metal complex on the PPy surface through π-π* interaction and coordination of Co(III) with PPy nitrogen atoms protect the Co(III) complex from getting demetallated in strongly acidic medium. Additionally, Co-complex, which is a square planar complex, provides open coordination sites for PPy nitrogen. Thus, PPy nitrogen easily interacts with the cobalt center holding it tightly in a matrix and stabilize the complex in acidic medium, which would account for the high stability of the thin film, effective ion penetration and/or electron transport and leads to a higher capacitance.

**Figure 3:**
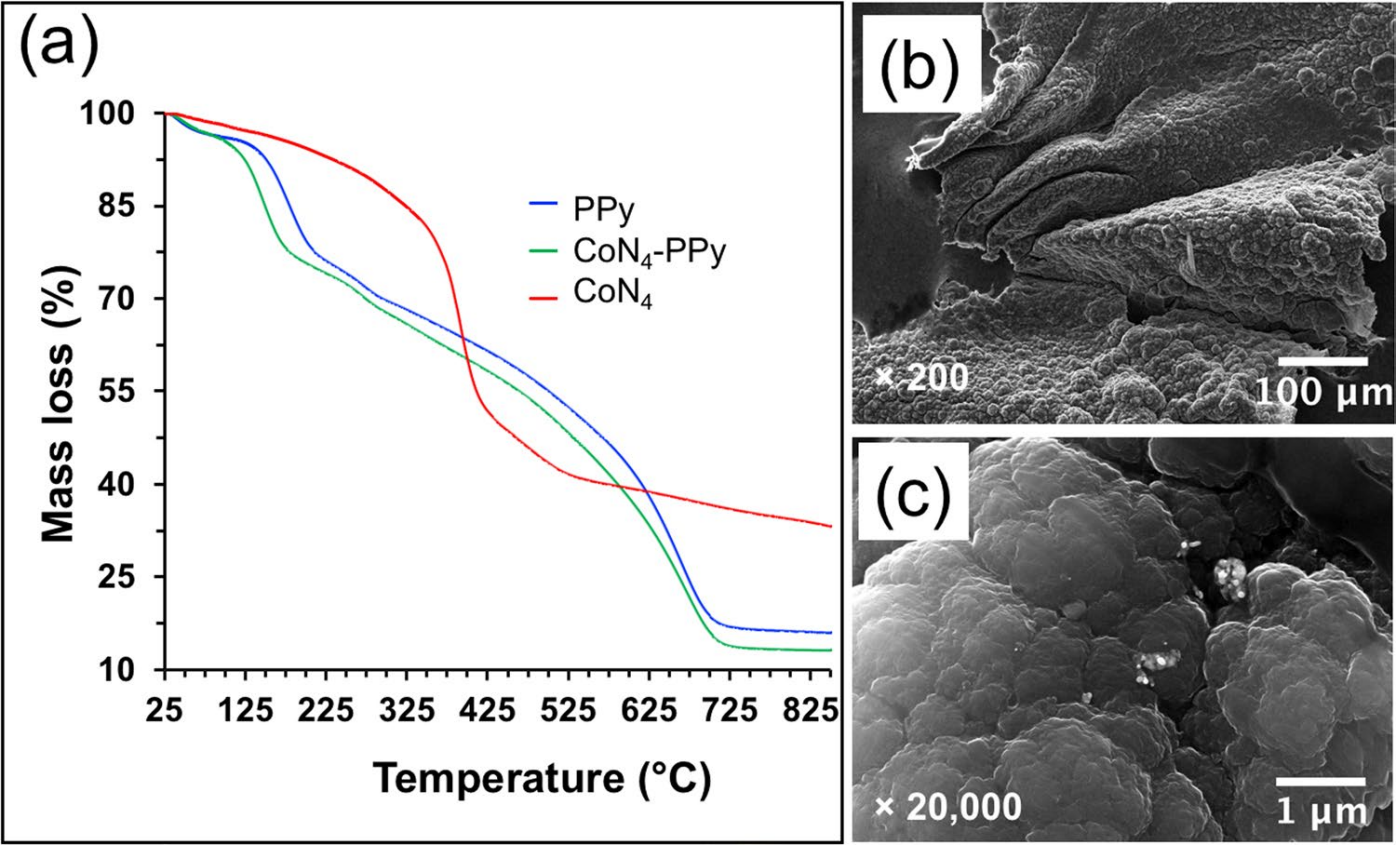
(**a**) TGA of PPy, CoN_4_-PPy and CoN_4_ thin films. SEM images of CoN_4_-PPy thin film at (**b**) x200 and (**c**) x20,000 magnifications.

Further characterization of CoN_4_-PPy was performed using thermogravimetric analysis (TGA) to study the thermal behavior of the thin films (Fig. 3a). The TGA profile of PPy and CoN_4_-PPy are similar with changes in the onset temperatures of degradation. There are three zones of thermal degradation. The first zone represents the initial loss in mass due to water desorption during heating. The second zone starts around 130 °C for PPy and indicated degradation of the pyrrolic backbone. CoN_4_-PPy began degradation at 105 °C, which was due to modification of PPy with CoN_4_. PPy retained more mass loss than CoN_4_-PPy, which is due to the increased presence of polypyrrole in the sample. It has been shown that higher amounts of PPy retain more mass after thermal degradation compared to materials that have decreased concentration of PPy, and, thus, the reason for greater mass loss in CoN_4_-PPy.

Surface morphology of CoN_4_-PPy was conducted using scanning electron microscope (SEM) and images were observed at x200, and x20,000 magnifications (Fig. 3b,c, respectively). The imaging shows uniform film surface with cauliflower-like clusters throughout, which is commonly observed in PPy materials (Fig. 3b)^43^. At higher magnification, the appearance of white clusters becomes noticeable (Fig. 3c). They are attributed to the dispersion of CoN_4_ on PPy film during electrochemical deposition, as is observed in other modified PPy materials^44^. To observe the distribution of Co complex on the surface of CoN_4_-PPy thin film, energy dispersive X-ray spectroscopy (EDS) elemental mapping on the scanning transmission electron microscopy (STEM) was performed (Supplementary Fig. S4a–e). Fig. S4a is the STEM image of CoN_4_-PPy thin films and Fig. S4b–e are the corresponding mapping images for C, N, O, and Co elements. The results illustrate uniform distribution of Co complex throughout the surface of the CoN_4_-PPy thin films despite of its low concentration. The EDS spectra of CoN_4_-PPy thin film is shown in Fig. S4f, which revealed the presence of C, N, O, and Co. Further, the three-dimensional (3-D) surface morphology, topography, and surface area of the thin film studied with 3-D laser scanning microscopy showed the uniformity of the sample without any cracks and resulted in the total surface area higher by 5.58 times than the initial or the blank surfaces for the scan areas (Supplementary Fig. S5 and Table S2).

### Electrochemical studies (Supercapacitive properties)

#### Effect of different electrolytes on supercapacitance

**Figure 4:**
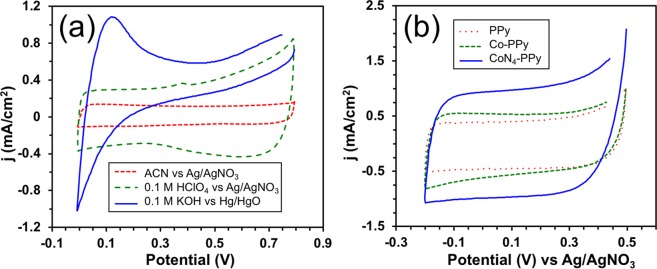
(**a**) CVs of PPy films on ACN, 0.1 M HClO_4_, and 0.1 M KOH electrolyte solutions, scan rate = 5 mV/s and potential scanning = 0 to 0.8 V. (**b**) CVs of PPy, Co-PPy and CoN_4_-PPy films in 0.1 M HClO_4_, scan rate = 10 mV/s and potential scanning = −0.2 to 0.5 V (versus Ag/AgNO_3_).

Cyclic voltammetry (CV) is a powerful tool in the study of capacitive effect of any electrode material where the choice of electrolyte solution plays an equally important role. Thus, to determine the proper electrolyte, CVs of PPy films electrode were conducted using organic and aqueous solvents. Acetonitrile (ACN) was chosen as an organic electrolyte whereas for aqueous electrolytes 0.1 M perchloric acid (HClO_4_) and 0.1 M potassium hydroxide (KOH) were selected. The result in Fig. 4a displays CVs obtained in different electrolytes when scanned at 5 mV/s in a potential window of 0.0 to 0.8 V. It can be observed that both ACN and 0.1 M HClO_4_ electrolytes showed great charge and discharge times for PPy films. In ACN, PPy films showed a rectangular type voltammogram with a maximum current density of 750 µA/cm^2^. The PPy film in ACN was being reduced (discharged) from 0.8 to 0.7 V (versus Ag/AgNO_3_), as indicated by the rapidly decreasing current, as negatively charged ions in the electrolyte solution left the film causing the film to act as an insulator. The film was fully discharged at this point and remained in its reduced state until the potential was decreased to 0.0 V (versus Ag/AgNO_3_) at which point the applied scan rate was reversed and began to increase the applied potential.

The PPy film was further being oxidized (charged) from 0.0 to 0.1 V (versus Ag/AgNO_3_), as indicated by the rapidly increasing current, as negatively charged ions in the electrolyte solution entered the film causing the film to act as a conductor^45^. The PPy film in ACN remained in its oxidized state as the potential increased and reached 0.8 V (versus Ag/AgNO_3_). A different phenomenon was observed with PPy films in 0.1 M HClO_4_. Like PPy films in ACN, PPy films in acidic media cycle through both a reduced (discharged) state and an oxidized state (charged). However, unlike PPy films in ACN, PPy films in acidic media never reached a full charge or discharge state; rather, the current started to rapidly increase at about 0.4 V (versus Ag/AgNO_3_) as the film was being oxidized until it reached a maximum current density at 0.8 V (versus Ag/AgNO_3_). This was attributed to a set of redox reaction that occured at the films surface indicating that PPy films in acidic media gain charge through both electrostatic forces as well as pseudocapacitative mechanisms^46^. The differences observed in the two media occurred due the nature of the electrolyte solution (aqueous versus organic). In aqueous solvents, their higher ionic conductivity and non-corrosiveness are believed to make them more advantageous than the organic electrolytes^47^. Furthermore, their low cost and lower toxicity benefits their widespread use. When comparing acidic versus alkaline electrolyte, 0.1 M HClO_4_ was a much better choice for supercapacitor application as seen in Fig. 4a. Redox reactions were observed in the 0.1 M KOH solution at the intense peak at 0.115 V (versus Hg/HgO), which could be due to the redox activity of PPy^48^. The presence of this peak has also been associated with a combination of changes in electronic and ionic conductivity as PPy is transitioning from an insulator to a conductor^48^. From these observations, 0.1 M HClO_4_ was chosen as the electrolyte solution for additional supercapacitor testing.

#### Effect on supercapacitance of different PPy films

The voltammogram responses of PPy film, PPy film with cobalt acetate (Co-PPy), and CoN_4_-PPy film were obtained in 0.1 M HClO_4_ electrolyte at 10 mV/s in a potential range of −0.2 to 0.5 V (versus Ag/AgNO_3_). The results, depicted in Fig. 4b, indicate that all three samples retained a characteristic rectangular type voltammogram suitable for supercapacitance with significant high magnitude of current density. Each film also accumulated charge quickly and dissipated it relatively quickly. CoN_4_-PPy film exhibited a noticeable increase in current density compared to PPy and Co-PPy films. The current density almost doubled from 0.539 mA/cm^2^ in Co-PPy to 0.979 mA/cm^2^ in CoN_4_-PPy at 0.110 V (versus Ag/AgNO_3_).

#### Effect of scan rate

**Figure 5:**
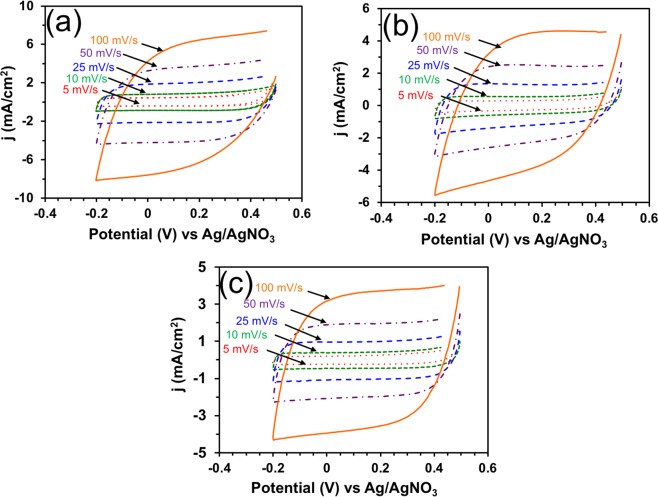
CVs of (**a**) CoN_4_-PPy film (**b**) Co-PPy film and (**c**) PPy film in 0.1 M HClO_4_ at increasing scan rates from 5 to 100 mV/s. Potential scanning = −0.2 to 0.5 V (versus Ag/AgNO_3_).

The CVs of PPy films were further performed at increasing scan rates from 5 to 100 mV/s (Fig. 5a-c). It was observed that the scan rate had a profound effect on the amount of current density observed. As the scan rate was increased, the current density also increased for each PPy film in 0.1 M HClO_4_. Another interesting effect observed was the decreasing rate of charge accumulation and dissipation as the scan rate was increased. This was observed as the shape of the CVs became less rectangular-shaped as the scan rate was increased. From these plots, the CoN_4_-PPy (Fig. 5a) exhibited the highest current density followed by Co-PPy (Fig. 5b) and PPy (Fig. 5c) films, respectively.

From the CV of each film, the specific capacitance (*C*_*g*_) for each of the three films was calculated by using the relationship between average current density (*i*_*avg*_), scan rate (*ν*) and the mass (*m*) of the films (eq. 1). This equation can further be equated with respect to charge (*Q*) and applied potential (*V*). The *C*_*g*_ of each film was calculated at scan rates from 5 to 100 mV/s and is given in Table 1.

1 $${C}_{g}=\frac{{i}_{avg}}{v\cdot m}=\frac{Q}{V\cdot m}$$

**Table 1:** Specific capacitance (*C*_*g*_) of PPy, Co-PPy, and CoN_4_-PPy film at increasing scan rate.

*Scan rate*	*Power (W) (* × ^*10−5*^*)*			*i*_*avg*_ *(mA/cm* ^*2*^ *) (* ^× *10−5*^ *)*			*C*_*g*_ *(F/g)*		
	PPy film	Co-PPy	CoN_4_-PPy	PPy film	Co-PPy	CoN_4_-PPy	PPy film	Co-PPy	CoN_4_-PPy
*5 mV/s*	4.97	8.44	1.51	6.21	1.05	2.16	216.1	351.8	721.9
*10 mV/s*	8.64	1.51	2.40	1.08	1.89	3.43	180.1	314.9	571.9
*25 mV/s*	1.87	3.32	5.10	2.34	4.15	7.29	156.0	276.6	486.4
*50 mV/s*	3.45	6.01	8.66	4.32	7.52	1.24	144.0	250.8	412.7
*100 mV/s*	6.25	1.07	1.29	7.81	1.34	1.84	130.2	223.1	306.2

Conditions: scan rate from 5 to 100 mV/s, 0.1 M HClO_4_ electrolyte solution, 25 °C, Ag/AgNO_3_ reference electrode.

A maximum specific capacitance was calculated to be 216.1, 351.8 and 721.9 F/g for PPy, Co-PPy and CoN_4_-PPy, respectively. The high specific capacitance of CoN_4_-PPy is greater than that of a Co hydroxide nanowire supported on exfoliated graphite oxide (610 F/g)^20^ and Mn and Co oxide nanowire array (480 F/g)^14^. Apart from this, the maximum specific capacitance exhibited by CoN_4_-PPy at 5 mV/s outstrips the values that were previously reported on PPy based electrode materials (Supplementary Table S3). There are several reasons that can be cited for the increased specific capacitance for the thin films. Co in both cobalt acetate and complex (CoN_4_) are responsible for providing redox active sites. However, improved solubility of CoN_4_ compared to Co acetate in 0.1 M HClO_4_ electrolyte used for electrochemical studies increased the redox active sites in its film. This phenomenon can be explained by the fact that high amount of CoN_4_ was deposited on PPy film during the electrpolymerization process and this contributed to higher current density of CoN_4_-PPy. As seen in Fig. 4b, the effect of pseudocapacitance was diminished in the CoN_4_-PPy film compared to Co-PPy at lower potential. The shape of the voltammogram curve for Co-PPy deviated from the characteristic rectangular shape of supercapacitors due to charge-transfer interaction between the electrode surface and the acidic electrolyte^9^.

Another possible reason for the increase in the specific capacitance lies in the film preparation. There are two common preparation techniques that were mentioned earlier: chemical precipitation and electrochemical deposition. In the former method, problems of particle agglomeration and presence of a binder hinder the prepared materials^47^. As a result, the active surface area of the material can be decreased, which increases the internal resistance. This generates a lower specific capacitance, as observed in a Co oxide/graphene composite^12^. Electrochemical deposition method that was employed in this study dispersed CoN_4_ throughout the PPy film, which allows for great active surface area. Consequently, the material generated a high current density with a large specific capacitance.

Finally, unique redox property and the structure of the complex over other Co materials also enhances its ability to hold and maintain charge. Within the complex structure, CoN_4_ has multiple sites that can undergo reduction/oxidation and, consequently, hold charge. To begin, the Co metal itself acts as a site for charge accumulation. As a transition metal, it has the ability to be easily reduced to a Co(II) state during discharge. The amidomacrocyclic ligand structure also contains areas of potential reduction/oxidation. The four carbonyl groups (C=O) can be reduced during discharge to C-O^−^ and oxidized back to the carbonyl as the material is being charged. Furthermore, the benzene ring can be reduced. These multiple locations of reduction/oxidation attributed to high charge storage of CoN_4_-PPy.

We compared our result with some of the recent works that has been done in the supercapacitor fields. In our work, the maximum specific capacitance value obtained using CoN_4_-PPy film on GCE for supercapacitor in 0.1 M HClO_4_ was significantly higher for those obtained using GCE modified with (Ag)-PPy/Graphene and mesoporous polyaniline (M-PANI) films in 1 M KOH and 1 M H_2_SO_4_, respectively^18,49^. Further, with respect to other samples that employed PPy but different substrate other than GCE for electrode preparation also lacked higher supercapacitor activity than CoN_4_-PPy film^8,20,50–53^. It was also found that some electrode materials were fabricated using Pt foil substrate, which use expensive and rare Pt metal^52^. Recently, some groups were successful to achieve specific capacitance value higher than the one we reported in this work. However, in their work reduced graphene oxide (RGO) and metal oxides were used for electrode making in Ni foam substrate^8,13^. Our experiments use Co complex, PPy and GCE substrate to achieve high specific capacitance, which was not reported elsewhere thus far.

**Figure 6:**
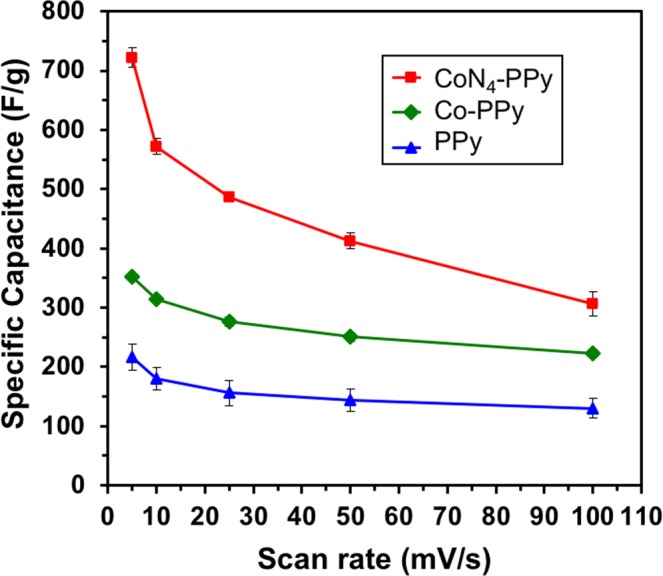
Variation of specific capacitance of PPy, Co-PPy and CoN_4_-PPy films with scan rate in 0.1 M HClO_4_ electrolyte.

The specific capacitance values were plotted with respect to scan rate as given in Fig. 6. The specific capacitance of films decreased quickly with scan rate up to 25 mV/s. At higher scan rates, the specific capacitance did not decrease as quickly, which showed a loss of dependence of the specific capacitance on increasing scan rate. From this plot, it is obvious that CoN_4_-PPy exhibited a much greater specific capacitance than the other films. Co-PPy had a larger specific capacitance than PPy, which showed how modification of PPy films can enhance its performance. However, the ability of CoN_4_ to provide more redox active centers for increased charge storage revealed that the nature of Co metal used (Co salt versus a Co catalyst) has a dramatic effect on capacitance.

GCD tests were performed at different current densities for CoN_4_-PPy in 0.1 M HClO_4_ electrolyte solution (Supplementary Fig. S7a). Asymmetrical charge and discharge curves were seen, indicating pseudo-capacitance behaviors of the CoN_4_-PPy thin film^54^. Additionally, the small IR drops, due to the electrode’s internal resistances, at the beginning of the discharge processes in the given current densities, were responsible for the non-linear discharge curves. These negligible IR drops can also explain conductivity and improved charge efficiency^54,55^. The specific capacitances values were measured at different current densities from GCD curves using eq. (2).

2 $$Cs=\,\frac{It}{{\rm{\Delta }}Vm}$$

where, C_s_ is the specific capacitance (F/g), I is the discharge current (A), t represents discharge time (sec), ∆V is the potential window (V), and m is the mass of the electro active material (g). As calculated from GCD curves using eq. (2), CoN_4_-PPy displayed the specific capacitances of 668, 564, 467, and 423 F/g at current densities of 0.45, 0.5, 1.5 and 2.5 mA/cm^2^, respectively. Fig. S7b shows the variation of specific capacitance at different current densities. The results show that the specific capacitance decreases when the applied current density increases. At higher current densities and thus higher polarization, less electrolyte will diffuse into the inner active sites, resulting in decreased specific capacitances^56,57^.

**Figure 7:**
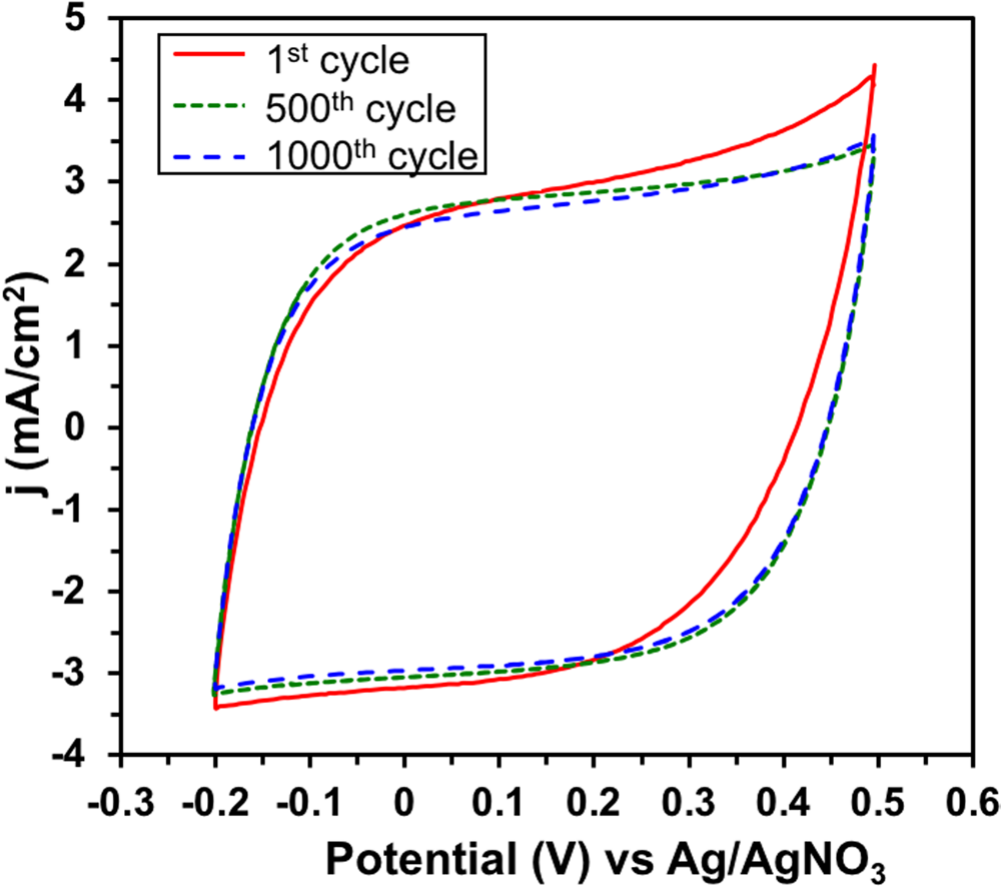
Stability of CoN_4_-PPy film in 0.1 M HClO_4_, scan rate = 25 mV/s and potential scanning = −0.2 to 0.5 V (versus Ag/AgNO_3_).

To demonstrate the electrochemical stability of CoN_4_-PPy, CVs were recorded up to 1000 cycles at 25 mV/s (Fig. 7). From the first cycle to the final, little change in current density was observed. The reason for the change could be due to small pseudocapacitance that is observed in the first cycle but later reduced in the 500^th^ and 1000^th^ cycle. During initial CV, any effect of pseudocapacitance becomes very evident. However, after longer cycles are performed, the degree of pseudocapacitance decreases and the charge storage mechanism shifts from a Faradaic to non-Faradaic process. Overall, CoN_4_-PPy was found to be electrochemically stable in strongly acidic media. This feature is advantageous for supercapacitors since the electrode material does not need to be replaced often and allows for uses of this material in remote locations^9^.

#### Effect of electrolyte concentration

The formation of a double layer within the electrode surface by electrolyte ion mobility depends on the electrolyte concentration^58,59^. Thus, electrolyte concentration has a significant effect on the electrical conductivity and the capacitance of the supercapacitor. Investigating this effect, we chose aqueous hydrochloric acid (HCl) electrolyte because it is one of the most widely used electrolyte solutions for supercapacitor applications. We studied the electrochemical behavior of the CoN_4_-PPy in 0.01, 0.1 and 1 M HCl by CV at a scan rate of 10 mV/s (Supplementary Fig. S6). As shown in Fig. S6, CoN_4_-PPy, in 0.1 M and 1 M HCl electrolytes showed ideal capacitive behavior displaying symmetrical rectangular shape voltammograms with no redox peaks; however, at low concentration (0.01 M), slight distortion of shape was observed which may be due to inadequate conductivity and mobility of ions for building-up the electric double layer. It was also observed that the current density of CoN_4_-PPy thin film increases gradually with the concentration of HCl electrolyte. In addition, the higher current density observed at higher electrolyte concentration (1 M HCl) indicated greater charge stored by the electrode. This behavior was developed due to high accumulation of electrolyte ion onto the double layer surface and their facile transport within the electrode layer. This observation was consistent with the previous findings^59,60^. Thus, it can be concluded that higher specific capacitance could be possible to achieve when higher electrolyte concentration is used.

#### Effect of using different electrochemical cell systems

Previous findings have established that capacitance value for the supercapacitor electrode material depends strongly on the type of electrochemical cell configurations^61^. In order to investigate this effect, along with three-electrode system, the supercapacitive performance studies of CoN_4_-PPy thin film, in two-electrode system, were also conducted. CV tests were carried out at a scan rate of 10 mV/s in 0.1 M HClO_4_ (Supplementary Fig. S8a,b). In can be seen from Fig. S8a that CoN_4_-PPy, in three-electrode system, showed a rectangular like shape indicating ideal capacitive behavior with reversibility. It also exhibited highest current density which indicated high specific capacitance of the electrode system. However, a different scenario was observed in case of two-electrode system. As shown in Fig. S8b, CoN_4_-PPy showed a narrower loop but still maintained the characteristic rectangular shape with a slight deviation. The narrower loop indicated low current density and consequently low specific capacitance of the electrode system. Based on the above result, the values of specific capacitance calculated for three- and two-electrode systems were found to be 533 F/g and 175 F/g, respectively. Similar results were most commonly observed for electrochemical supercapacitor materials^61,62^. The extremely high value of specific capacitance found in the case of three-electrode system compared to the two-electrode system illustrates the fact that the latter system only provides a good approximation of electrode performance in supercapacitors^61^.

#### GCD cyclic stability and electrochemical impedance spectroscopy (EIS) measurement

The GCD cyclic stability and specific capacitance retention of CoN_4_-PPy thin film were investigated at applied current density of 0.5 mA/cm^2^ for 500 cycles in 0.1 M HClO_4_ (Supplementary Fig. S9a). It was observed that CoN_4_-PPy retains 93% of the initial capacitance after 500 cycles. Commonly, poor cyclic stabilities were observed with electrically conducting polymer (ECP) films due to the fast degradation of the polymer chains, low electrochemical stabilities, and loss of active materials resulting from the continuous swelling/shrinkage and thus the formation of cracks during cycling^63^. However, past studies have proved that incorporating transition metal ions or complexes or oxides onto the conducting polymers effectively suppress the crack formation of polymeric thin film electrode during cycling and effectively enhanced the electrochemical performance^52,64^. Thus, we believe the inclusion of CoN_4_ into the PPy film could suppress the cracking of the PPy backbone during GCD cycling and increase its conductivity resulting in the high specific capacitance and improved cyclic stabilities. Additionally, cobalt metallic center in CoN_4_ can offer more active sites of faradic reaction and facilitate the charge transfer for CoN_4_-PPy thin film electrode^65,66^. Supplementary Fig. S9a also showed that CoN_4_-PPy thin film produced fairly stable specific capacitance after 350 cycles, except for the initial 7% decline in specific capacitance. This observed initial decline in specific capacitance may be some of the surface adsorbed CoN_4_ complex in CoN_4_-PPy thin film was either leached out or degraded onto the acidic electrolyte solution resulting in decrease of active surface area^67^. The overall improved cyclic stability may be attributed to the affinity of the metal-complex to bind with polypyrrole by π-π* interaction of benzene rings, counter-ion interactions, and morphology of the sample, which are ideal for easy electrons mobility and charge transfer^66,68^. Further, to study the possible presence of CoN_4_ or it’s degraded form into the electrolyte solution, UV-vis spectroscopic analysis of the electrolyte solution sample withdrawn after 100 cyclic electrochemical measurements in 0.1 M HClO_4_ was performed (Supplementary Fig. S10). However, the result showed that the concentration of the CoN_4_ or decomposition product of the sample was not detectable in an appreciable limit into the electrolyte. Electrochemical impedance spectroscopy (EIS) measurements were performed for CoN_4_-PPy film before and after 500 GCD cycles and the corresponding impedance (real and imaginary) values for both were obtained (Supplementary Fig. S9b). According to the Nyquist plots in Fig. S9b, semi-circles in the high frequency region, which corresponds to a charge-transfer resistances (R_ct_), vary slightly during cycling, further supporting its cycling stability.

## Conclusions

PPy films with CoN_4_ complex and a cobalt salt (Co(OAc)_2_) were successfully synthesized by electrochemical oxidation *via* electrochemical deposition. Acidic media proved to be the optimal electrolyte choice over alkaline or organic solvents. The unique redox property and solubility of CoN_4_ compared to Co(OAc)_2_, allowed CoN_4_ to accumulate inside PPy films to a greater extent leading to an increase in overall capacitance. CoN_4_-PPy had the highest specific capacitance value of 721.9 F/g in 0.1 M HClO_4_ at a scan rate of 5 mV/s. Moreover, excellent electrochemical stability was observed up to 1000 cycles in acidic media. GCD revealed low rate for charging or discharging, which indicated improved capacitance and charge storage. CoN_4_-PPy displayed excellent stability and high specific capacitance retention after 500 GCD cycles. The high specific capacitance and ease of synthesis for these films make them ideal electrode materials for supercapacitors.

## Methods

### General

Chemicals used in this study were purchased from Sigma-Aldrich, USA or Acros Organics, USA and were used as received unless otherwise noted. Pyrrole was distilled using calcium hydride as a drying agent. Nitrogen gas (ultra-high purity) was obtained from NLR Welding Supply Inc. Cyclic voltammetry (CV) studies were performed using a Pine Instrument (Grove City, PA) bipotentiostat (Model AFCBP1) at 25 °C. Galvanostatic charge-discharge (GCD) and electrochemical impedance spectroscopy (EIS) tests were performed using CHI660D electrochemical analyzer. Thermo Scientific K-Alpha X-ray Photoelectron Spectroscopy (XPS) system was utilized to obtain the XPS spectra. Scanning electron microscope (SEM) coupled with energy-dispersive X-ray spectroscopy (EDS) was carried out by using JEOL SEM (JSM 7000 F). Raman spectra were recorded using a Raman spectrometer (Horiba Jobin Yvon LabRam HR800, Edison, New Jersey). Thermogravimetric analysis (TGA) was performed with a Shimadzu DTG-50 thermal analyzer, where samples were heated from 25 to 850 °C at a heating rate of 10 °C/min. Three-dimensional (3D) surface morphology, topography and surface area were evaluated using a laser scanning confocal microscope (LSCM, VK-X260K, Keyence, USA). Samples were scanned using 100X lens and the scanning spot dimension was 108 μm × 145 μm. 3D measurement data collected were analyzed with Keyence’s Multi-File Analyzer software. UV-vis spectroscopy was performed using AGILENT (Varian) Cary 5000 Spectrophotometer, USA.

### Synthesis of ligand (N_4_) and Co(III) complex (CoN_4_)

Synthesis of the amidomacrocyclic ligand (N_4_) was conducted following a previously published procedure^69^ using 4,5-dichloro-*o*-phenylene diamine to generate the dichloro version of this ligand. Synthesis of the cobalt complex was performed as follows. In a 100-mL Schlenk flask containing a magnetic stir bar, N_4_ (200 mg, 0.45 mmol) was added and dissolved in 20 mL dry tetrahydrofuran (THF) by stirring under N_2_ atmosphere at 25 °C. After dissolution, the temperature was lowered to 0 °C by placing the flask in an ice bath. Once the solution was cooled, *n*-butyllithium (0.74 mL, 1.8 mmol, 2.5 M in hexanes) was added, followed by anhydrous cobalt (II) chloride (64 mg, 0.50 mmol). The reaction was slowly warmed to room temperature and allowed to stir overnight under N_2_ atmosphere. The reaction yielded a precipitate, which was exposed to air to allow the cobalt to oxidize from Co(II) to Co(III). The THF was removed under vacuum and the solid purple product **(**Co(III) complex) obtained was further dried under vacuum for 6 h. Yield: 78% (175 mg). The Co(III) complex was converted to the water insoluble form by addition of tetraphenylphosphonium chloride to generate CoN_4_^70^.

### Electrochemical deposition of films

The synthetic approach for the electrochemical synthesis of PPy thin films was reported earlier^71^. For the synthesis of PPy films, 20 mM of distilled pyrrole dried over calcium hydride was used. 10 mL solutions containing a 1:1 volumetric ratio of propylene carbonate and acetonitrile (ACN) and 50 mM of tetrabutylammonium hexafluorophosphate (TBAPF_6_) as the supporting electrolyte was prepared as the solution from which deposition was performed. The potential was scanned from 0.00 to 1.20 V at a scan rate of 100 mV/s for 25 cycles, which allowed ten minutes for deposition. After deposition, the film was washed with distilled water followed by an ACN wash and allowed to dry. In a similar manner, CoN_4_-PPy films were synthesized using 20 mM of pyrrole and 20 mM of CoN_4_ catalyst. A last set of films called Co-PPy films were deposited using 20 mM of pyrrole and 10 mM of cobalt acetate (Co(OAc)_2_). Following electrochemical deposition, the three films-PPy, CoN_4_-PPy and Co-PPy were tested for supercapacitance. All electrochemical synthesis used a three-electrode electrochemical cell. The working electrode was a glassy carbon electrode (GCE), the reference electrode was Ag/AgNO_3_ (AgNO_3_ in ACN) and the counter electrode was a platinum (Pt) wire.

### Electrochemical studies

CV was performed with a potential range from 0.8 to 0 V at varying scan rate of 5 to 100 mV/s, unless otherwise noted. GCD tests were performed within a potential range from −0.2 to 0.4 V at different current densities (0.45, 0.5, 1.5 and 2.5 mA/cm^2^)_._ EIS measurements were obtained at an amplitude voltage of 5 mV from 1 Hz to 1 MHz in 0.1 M HClO_4_. A three-electrode system using a GCE working electrode, Ag/AgNO_3_ reference electrode in acidic media and Hg/HgO in basic media and platinum counter electrode was used when performing CV, GCD, and EIS. In case of two-electrode system in acidic condition, electrochemical cell consists of GCE as working electrode and Pt wire as both reference and counter electrode. All electrochemical tests were performed at room temperature. Within the electrochemical cell, the electrolyte was purged with N_2_ for at least 30 min prior to use. Voltammograms presented show the average of three trials.

### X-ray Photoelectron spectroscopy

XPS analysis was carried out with a Thermo Scientific Model K-Alpha XPS instrument using monochromatic Al Kα radiation (1486.7 eV) with the X-ray spot size 200 μm for each sample. The base pressure in the analysis chamber was typically 1 × 10^−9^ mbar. Samples were mounted to the sample platten using double-sided tape. All spectra were collected with the charge neutralization flood gun turned on. The typical pressure during the analysis with the flood gun on was 2 × 10^−7^ mbar. The collected data were processed using the Thermo Scientific Advantage XPS software package. Spectral charge correction was performed using the main C 1 s peak due to hydrocarbon (C-C/C-H bonds) set to 284.8 eV. Mixed Gaussian-Lorentzian peak shapes and a Shirley/Smart type background subtraction were utilized in the peak analysis/fitting.

### Raman spectroscopy

Raman spectra were recorded using a Raman spectrometer (Horiba Jobin Yvon LabRam HR800, Edison, New Jersey) occupied by He-Ne laser (17 mW) with wavelength of 784 nm and three Olympus BX-51 lenses with 100x micro objectives magnitude connected to a Peltier-cooled CCD camera. The spectra were collected using 600-line∕mm grating with the same acquisition time. In all measurements, the Raman spectrometer was calibrated using the Si-Si Raman signal, which is located at a 521 cm^−1^ Raman shift, and conducted at room temperature.

## Supplementary information

Supplementary information
