# Supplementary material for: Simultaneous Electrochemical Deposition of Cobalt Complex and Poly(pyrrole) Thin Films for Supercapacitor Electrodes
Source: Sci Rep. 2019 Apr 4;9:5650. doi: 10.1038/s41598-019-41969-6 (PMC6449390; doi:10.1038/s41598-019-41969-6)
Supplement: Supplementary file 1 — Supplementary information [file 41598_2019_41969_MOESM1_ESM.pdf]

Supplementary Information

Simultaneous Electrochemical Deposition of Cobalt Complex and Poly(pyrrole) Thin Films for Supercapacitor Electrodes

Charlette M. Parnell[1+], Bijay P. Chhetri[1+], Travis B. Mitchell[1], Fumiya Watanabe[2], Ganesh Kannarpady[2], Ambar B. RanguMagar[1], Huajun Zhou[3], Karrer M. Alghazali,[2] Alexandru S. Biris[2*], Anindya Ghosh[1*]

[1]*Department of Chemistry, University of Arkansas at Little Rock, 2801 South University Avenue, Little Rock, AR 72204, USA*

[2]*Center for Integrative Nanotechnology Sciences, University of Arkansas at Little Rock, 2801 South University Avenue, Little Rock, AR 72204, USA*

[3]*High-Density Electronics Center, University of Arkansas, Fayetteville, AR 72701, USA*

[+]These authors contributed equally to this work

*Corresponding Authors
Email: asbiris@ualr.edu, Phone: (501) 683-7458, (501) 683-7601
E-mail: axghosh@ualr.edu, Phone: (501) 569-8827, Fax: (501) 569-8838

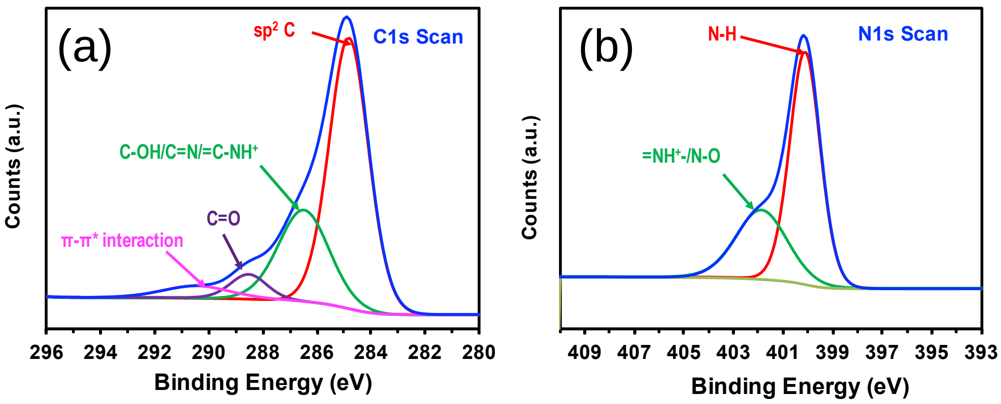

Figure S1. XPS narrow scans of **(a)** carbon (C1s) and **(b)** nitrogen (N1s) atoms present in PPy film.

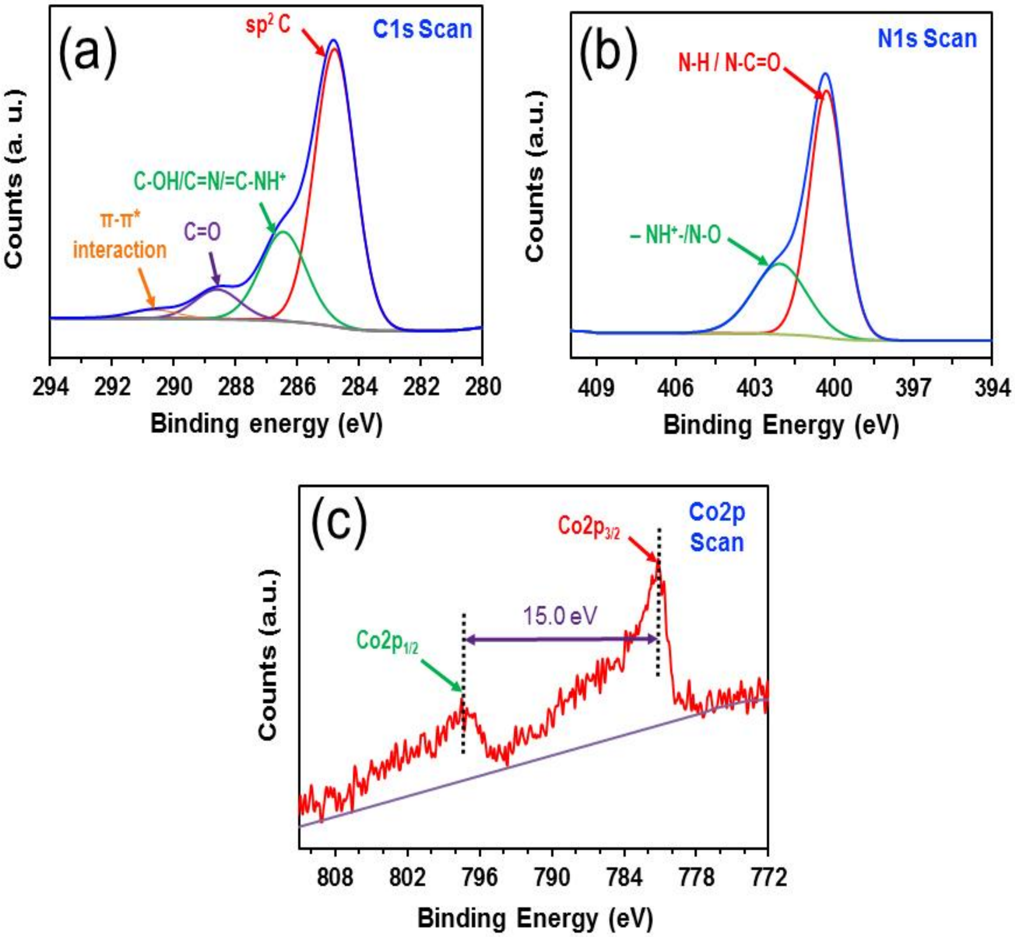

Figure S2. XPS narrow scans of **(a)** carbon (C1s), **(b)** nitrogen (N1s), and **(c)** cobalt (Co2p) present in CoN_4-PPy film after exposure with 0.1 M $HClO_4$.

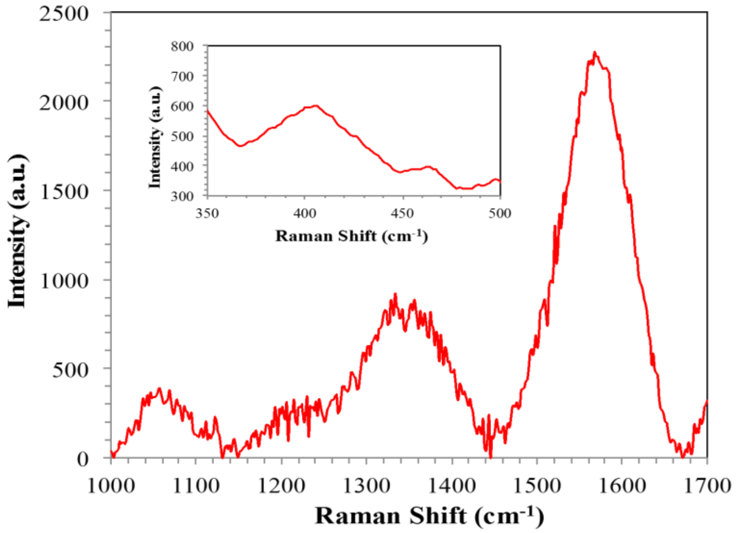

Figure 3. Raman spectrum of **2**-PPy film.

Figure S3. Raman spectrum of CoN_4-PPy film after exposure with 0.1 M $HClO_4$.

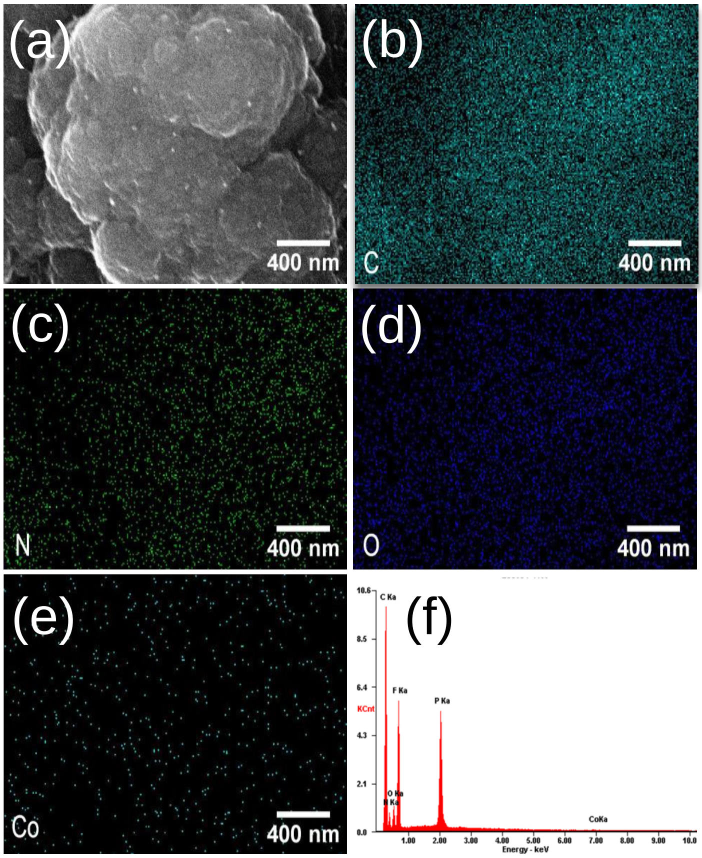

Figure S4. EDS elemental mapping images **(b-e)** showing the distribution of C, N, O, and Co elements in CoN_4-PPy thin film obtained from STEM image shown on figure **(a)** and EDS spectra of CoN_4-PPy **(f)**.

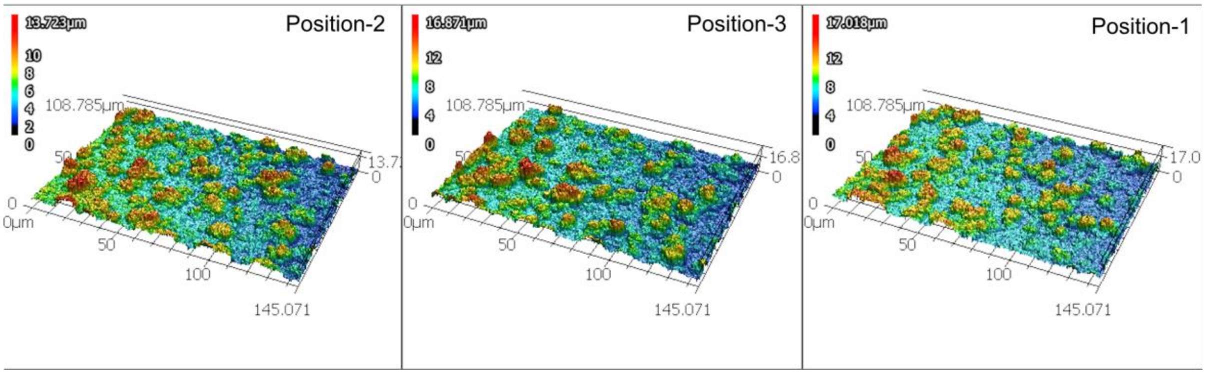

Figure S5. 3-Dimensional laser scanning microscopy images of CoN_4-PPy thin film obtained at three different positions.

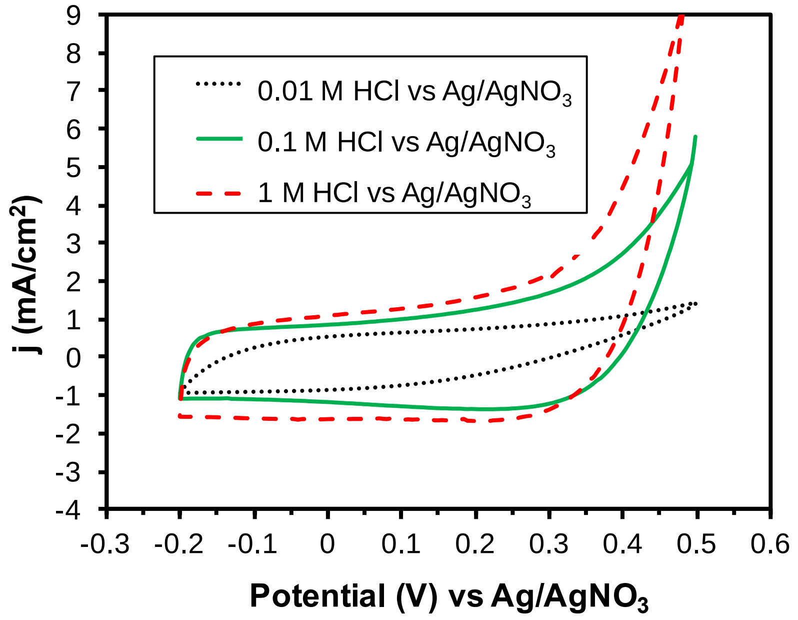

Figure S6. CVs of CoN_4-PPy film in 0.01, 0.1, and 1 M HCl electrolyte solutions, scan rate = 10 mV/s and potential scanning = -0.2 to 0.5 V (versus Ag/ $AgNO_3$).

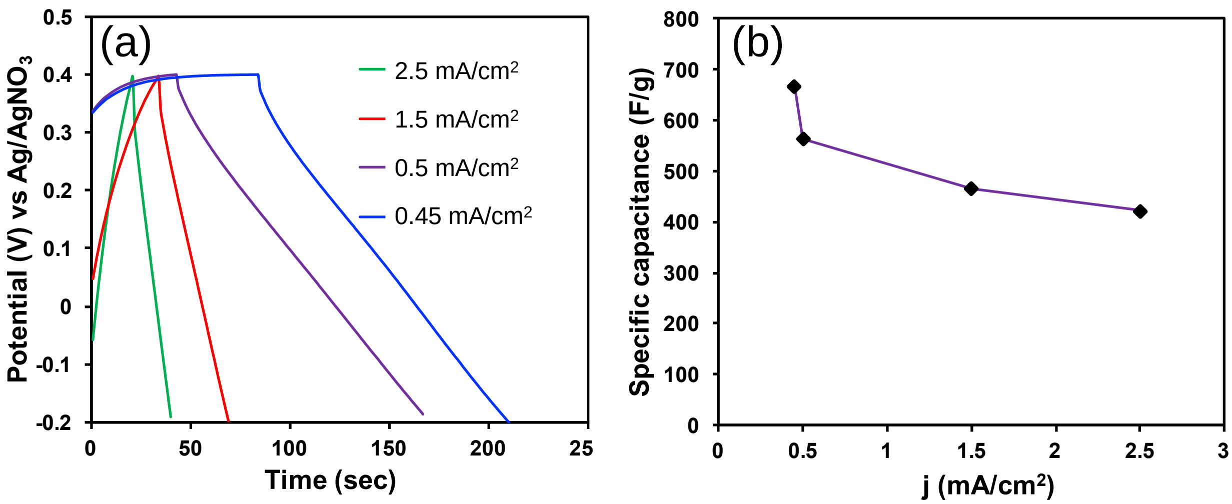

Figure S7. (a) GCD curves of CoN_4-PPy at different current densities and **(b)** Specific capacitance (F/g) of CoN_4-PPy as a function of current densities

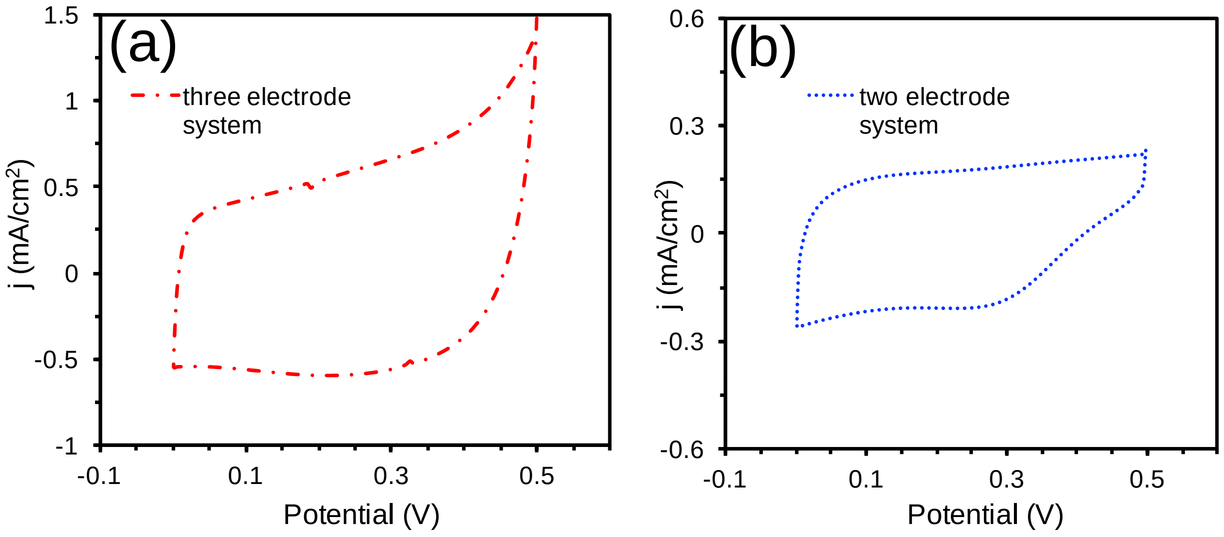

Figure S8. CVs at 10 mV/s of CoN_4-PPy thin film in three-electrode and two-electrode electrochemical cell system (electrolyte: 0.1 M $HClO_4$).

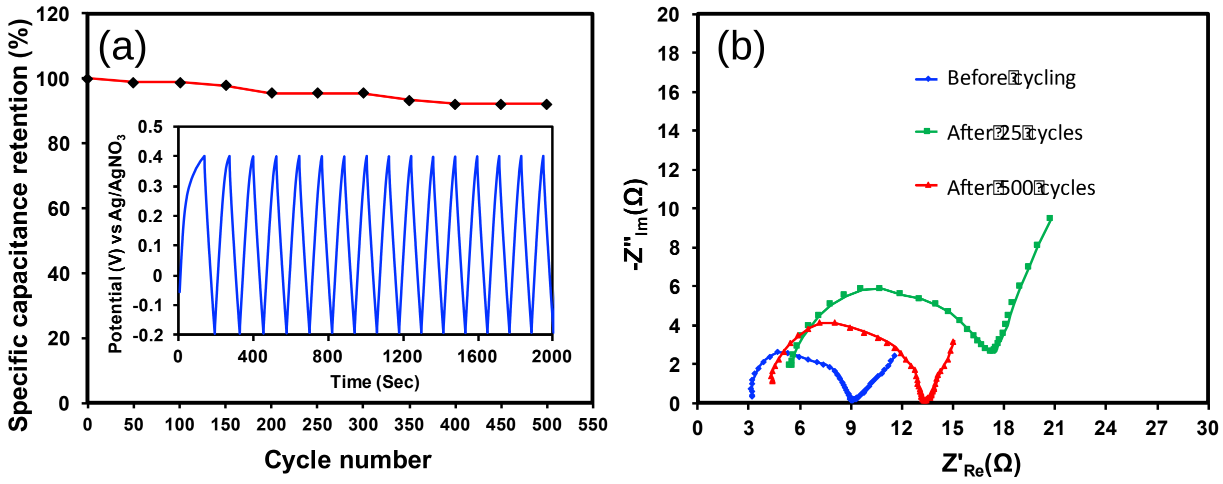

Figure S9. (a) Specific capacitance retention of CoN_4-PPy measured at 0.5 mA/cm^2 for 500 GCD cycles with the starting curves shown in the inset, **(b)** Nyquist plots of CoN_4-PPy before cycling and after 25 and 500 cycles.

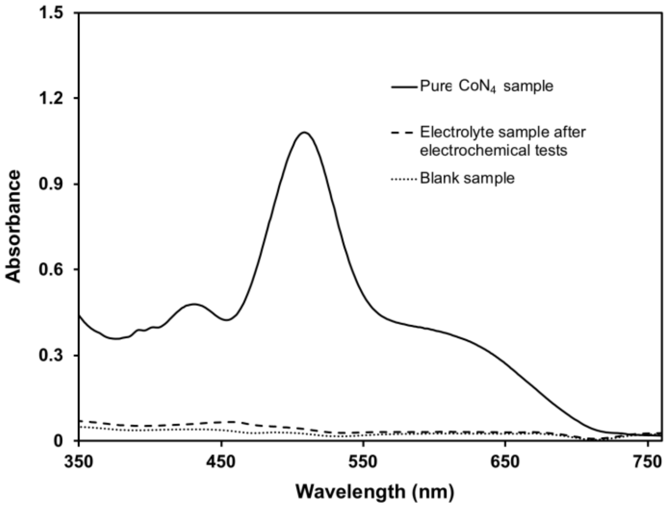

Figure S10. UV-vis spectra obtained for the acidic electrolyte sample after 100 electrochemical cyclic tests of CoN_4-PPy thin film in 0.1 M $HClO_4$. For comparison, UV-vis spectra of pure cobalt-complex (CoN_4) solution in acetonitrile and 0.1M $HClO_4$ as a blank sample were also obtained. Cobalt complex (CoN_4) was not detected in an appreciable limit in the electrolyte solution of the CoN_4-PPy thin film.

Table S1. Elemental identification and quantification from XPS analysis of CoN_4-PPy

Name	***Peak BE***	***FWHM eV***	***Area (P) CPS.eV***	***Atomic %***
C1s	284.87	3.50	680830.90	61.00
O1s	532.27	3.56	237330.88	8.80
N1s	400.09	3.41	152375.58	8.80
Co2p	781.80	1.92	1741.86	0.21
F1s	686.10	3.04	576633.78	17.06

Table S2. Total surface area of CoN_4-PPy thin film for the scan area (108 μm x 145 μm) obtained using 3-Dimensional laser scanning microscopy.

**Surface area (μm^2)**	**Surface area (μm^2)**	**Surface area (μm^2)**	**Mean-Surface area (μm^2)**	**Standard Deviation**
Position-1: 87898.26	Position-2: 87356.42	Position-3: 88772.15	88008.94	714.32

Table S3. Comparative study of CoN_4-PPy film with other PPy and related electrode materials for supercapacitors applications.

**Electrode material**	**Synthetic method**	**Electrolyte**	**Specific capacitance (F/g)**	**Ref.**
PPy nanosheets	Potentiodynamic deposition	0.5 M H_2SO_4	586 F/g at 2 mV/s	1
Self-assembled PPy film	interfacial polymerization	1 M $NaNO_3$	261 F/g at 25 mV/s	2
PPy/carbon aerogel	Chemical oxidative polymerization	6 M KOH	373 F/g at 5mV/s	3
PPy/MWCNT	Chemical polymerization	1 M H_2SO_4	320 at 5mV/s	4
MoS_2/PPy-n-ultrathin film	*in-situ* polymerization	1 M KCl	695 F/g at 10 mV/s	5
PPy-Ru (12)	Electrochemical polymerization	1 M H_2SO_4	458 F/g at 5 mV/s	6
PPy/NiCoHCF	Anodic polymerization	0.50 M $LiClO_4$/EC: DMC	453 F/g at 50 mV/s	7
Co_3O_4@polypyrrole/MWCNT	*in-situ* microemulsion polymerization	6 M KOH	615 F/g at 10 mV/s	8
PPy/FeO@CVO	*in-situ* chemical oxidative polymerization	1 M KOH	655 F/g at 50 mV/s	9
Ag-PPy/Graphene composite	*in-situ* oxidative polymerization	1 M KCl	474 F/g at 5mV/s	10
**CoN_4-PPy film**	**electropolymerization**	**0.1 M $HClO_4$**	**721.9 F/g at 5 mV/s**	**This work**

Note: PPy: Polypyrrole, MWCNT: Multiwalled carbon nanotube, MoS_2: Molybdenum disulfide, Ru: Ruthenium, NiCoHCF: Nickel-cobalt hexacyanoferrate, Co_3O_4: Cobalt (II, III) oxide, FeO: Iron oxide, CVO: Cobalt vanadium oxide, Ag: Silver.

References:

(1) Dubal, D. P.; Lee, S. H.; Kim, J. G.; Kim, W. B.; Lokhande, C. D. Porous polypyrrole clusters prepared by electropolymerization for a high performance supercapacitor. *J. Mater. Chem.* **2012**, *22*, 3044–3052.

(2) Yang, Q.; Hou, Z.; Huang, T. Self-assembled polypyrrole film by interfacial polymerization for supercapacitor applications. *J. Appl. Polym. Sci.* **2014**, *132*, 41615.

(3) An, H.; Wang, Y.; Wang, X.; Zheng, L.; Wang, X.; Yi, L.; Bai, L.; Zhang, X. Polypyrrole/carbon aerogel composite materials for supercapacitor. *J. Power Sources* **2010**, *195*, 6964–6969.

(4) Khomenko, V.; Frackowiak, E.; Beguin, F. Determination of the specific capacitance of conducting polymer/nanotubes composite electrodes using different cell configurations. *Electrochim. Acta* **2005**, *50*, 2499–2506.

(5) Tang, H.; Wang, J.; Yin, H.; Zhao, H.; Wang, D.; Tang, Z. Growth of polypyrrole ultrathin films on MoS_2 monolayers as high-performance supercapacitor electrodes. *Adv. Mater.* **2015**, *27*, 1117–1123.

(6) Zhou, Y.; Xie, Y. Capacitive performance of ruthenium-coordinated polypyrrole. *New J. Chem.* **2017**, *41*, 10312–10323.

(7) Ensafi, A. A.; Ahmadi, N.; Rezaei, B. Electrochemical preparation and characterization of a polypyrrole/nickel-cobalt hexacyanoferrate nanocomposite for supercapacitor applications. *RSC Adv.* **2015**, *5*, 91448–91456.

(8) Ramesh, S.; Haldorai, Y.; Kim, H. S.; Kim, J.-H. A nanocrystalline Co_3O_4@polypyrrole/MWCNT hybrid nanocomposite for high performance electrochemical supercapacitors. *RSC Adv.* **2017**, *7*, 36833–36843.

(9) Maitra, A.; Das, A. K.; Karan, S. K.; Paria, S.; Bera, R.; Khatua, B. B. A mesoporous high-performance supercapacitor electrode based on polypyrrole wrapped iron oxide decorated nanostructured cobalt vanadium oxide hydrate with enhanced electrochemical capacitance. *Ind. Eng. Chem. Res.* **2017**, *56*, 2444–2457.

(10) Dhibar, S.; Das, C. K. Silver nanoparticles decorated polypyrrole/graphene nanocomposite: A potential candidate for next-generation supercapacitor electrode material. *J. Appl. Polym. Sci.* **2017**, *134* (16), 44724-44738.
